# ﻿Two new species of the genus *Brunettia* Annandale, 1910 (Diptera, Psychodidae, Psychodinae) from China

**DOI:** 10.3897/zookeys.1259.153178

**Published:** 2025-11-11

**Authors:** Shuai-Lai Yang, Xin-Ming Yin, Yu-Qiang Xi

**Affiliations:** 1 State Key Laboratory of Wheat and Maize Crop Science/Henan International Laboratory for Green Pest Control; Department of Entomology, Henan Agricultural University, No. 95 Wenhua Road, Jinshui District, Zhengzhou 450003, Henan Province, China Henan Agricultural University Zhengzhou China

**Keywords:** *
Brunettia
*, Brunettiini, China, identification key, moth flies, new species, Psychodidae, taxonomy

## Abstract

Two new species of the genus *Brunettia* Annandale, 1910, collected from China, are described as new to science: *Brunettia zunyiensis***sp. nov.** and *Brunettia sinensis***sp. nov.** An updated key to the species of *Brunettia* from China is presented.

## ﻿Introduction

Psychodidae species are diverse and abundant, with about 3190 described species known worldwide (Munstermann 2019). The subfamily Psychodinae is the most species-rich in Psychodidae, with about 160 genera and 2000 species (Munstermann 2019). Following the classification by Kvifte (2018): *Brunettia* Annandale, 1910 belongs to Psychodinae, tribe Brunettiini. The genus can be diagnosed based on the following characters: species with 13 flagellomeres; wings with well-developed costal and anal areas; R_s_ pectinate with radial fork (R_2+3_) basal to medial fork (M_1+2_); R_5_ ending at tip; wing membrane with bristles; tergite 9 and gonocoxite without bristles; gonostyle with pair of long bristles at apex; and aedeagus symmetrical (Bravo 2006).

*Brunettia* has four subgenera: *Maurobrunettia* Duckhouse, *Campanulobrunettia* Duckhouse, *Brunettia* Annandale and *Neobrunettia* Bravo & Amorim (Bravo and Amorim 1995; Bravo 2006). The subgenus Maurobrunettia is characterized by the scape elongated, longer than posterior margin of eyes, and pedicel elongated, longer than wide, pyriform or reniform. The subgenus Campanulobrunettia is characterized by the ascoids palmate, with many branches; gonostylus flattened and enlarged, paddle shaped; and tenacula bell-tipped. The subgenus Neobrunettia is similar to the subgenus Brunettia, and they share the following common characteristics: ascoids not palmate, of other shapes; gonostylus never paddle-shaped; and tenacula clavate or uniseriate; however, the subgenus Neobrunettia has the wing vein R_1_ with a rupture in the basal fourth; Sc unsclerotinized, present as only a hair line; and the subgenus Brunettia has wing vein R_1_ without a rupture; and S_c_ strongly sclerotized (Bravo 2006).

There are more than 110 known species of *Brunettia* distributed widely in the world except in the Nearctic region (Kvifte and Wagner 2017). About 36 species are distributed in the Oriental and Palearctic regions (Annandale 1908; Brunetti 1908, 1911; Freeman 1951; Tokunaga and Komyo 1955; Satchell 1958; Tokunaga 1959; Quate 1962, 1965; Ipe et al. 1986; Huang and Chen 2001). Only six species are known in Taiwan Province, China: *B. lungjingensis* Huang & Chen, *B. autumna* Huang & Chen, *B. rotundior* Huang & Chen, *B. setiala* Huang & Chen, *B. subdisiunctio* Huang & Chen, and *B. albonotata* (Brunetti) (Lin and Chen 1999; Huang and Chen 2001).

In this study, two new species are described from China: *Brunettia zunyiensis* sp. nov. and *B. sinensis* sp. nov., both belonging to the subgenus Brunettia. A key to the known species of *Brunettia* from China is presented.

## ﻿Material and methods

The head, wings, and male genitalia were dissected separately, genitalia preparations were made by removing and macerating the apical portion of the abdomen in glacial acetic acid, then rinsing in distilled water before storage in glycerin-filled microvials. After examination, the head, wings and genitalia were all preserved in small tubes filled with glycerol. Specimens were examined and photographed using a Leica M205A stereomicroscope. Helicon Focus v. 7.0.2 was used for image stacking. Scale drawings were created based on the photographs, with partial structural details observed and refined under a stereomicroscope through adjustments and modifications before finalization. Image plates were post-processed with Adobe Photoshop CC 2019 Extended.

All measurements of adults are in millimetres (mm). Palpal proportions are calculated with the first segment as unit 1.

Specimens examined were deposited in the Entomological Museum of Henan Agricultural University (HAU), Zhengzhou. Terminology follows Kvifte and Wagner (2017) and Cumming and Wood (2017).

## ﻿Taxonomy

### ﻿Key to the species of *Brunettia* of China

**Table:** 

1	Eye bridge separated (Fig. 4A)	**2**
–	Eye bridge contiguous (Fig. 1A)	**4**
2	Eye bridge separated by more than 1 facet diameter	***B. albonotata* (Brunetti, 1908) (female)**
–	Eye bridge separated only by 0.5 or at most by 1 facet diameters	**3**
3	Wing vein R_5_ ending at wing apex; ejaculatory apodeme rod-shaped, surstylus biformed, tenacula bent	***B. subdisiunctio* Huang & Chen, 2001**
–	Wing vein R_5_ ending slightly below wing apex (Fig. 4C); ejaculatory apodeme not rod-shaped (Fig. 4E); surstylus short stick-shaped, tenacula not bent	***B. zunyiensis* sp. nov.**
4	Wing with humeral and anal regions expanded	**5**
–	Wing with humeral and anal regions normal	**6**
5	Ratio of wing length to width 1.91; ejaculatory apodeme broad at apex, 2.2× length of aedeagus	***B. sinensis* sp. nov.**
–	Ratio of wing length to width 1.59; ejaculatory apodeme pointed at apex, 1.5× length of aedeagus	***B. lungjingensis* Huang & Chen, 2001**
6	Surstylus with 3 tenacula forms (short and straight, short and bent, long and bent); parameres basal style round	***B. setiala* Huang & Chen, 2001**
–	Surstylus with 2 tenacula forms (short and straight, long and bent); parameres basal style narrow or not round	**7**
7	Eye bridge rounded at apex; parameres short, about 1.1 × length of apical shafts of aedeagus, base of parameres broad	***B. rotundior* Huang & Chen, 2001**
–	Eye bridge square at apex; parameres long, about 1.3 × length of apical shafts of aedeagus, base of parameres slender	***B. autumna* Huang & Chen, 2001**

#### ﻿ Brunettia sinensis sp. nov.

Taxon classificationAnimaliaDipteraPsychodidae

C4A5457C-ADE7-591F-AF11-F8343568F863

https://zoobank.org/7C95B6AE-8238-4248-ACB6-E877A5FE12F2

Figs 1A–F, 2A, B

##### Type material.

***Holotype*** • 1 ♂, **China**, Jilin, Changchun, Jingyuetan, 143°46'53"N, 125°27'48"E, 246 m, 2022.VIII.16, leg. Yuqiang Xi; ***Paratypes***: • 1 ♂, China, Fujian, Fuding, Taimu Mt., Jinfeng Temple, 27°06'48"N, 120°10'15"E, 591 m, 2024. IV. 26–2024. V. 10, leg. Malaise Trap. • 6 ♂♂, China, Fujian, Fuding, Huangren Village, Huangren Mt., 27°23'49"N, 120°18'58"E, 260 m, 2024. IV. 26–2024. V. 10. leg. Malaise Trap; • 1 ♂, China, Fujian, Fuzhou, Fuzhou National Forest Park, 26°09'41"N, 119°17'08"E, 230 m, 2024. VI. 23, leg. Rong Huang; • 2 ♂♂, China, Fujian, Fuzhou, Fuzhou National Forest Park. 2024. VI. 30, 26°09'51"N, 119°17'08"E, 253 m, leg. Shuailai Yang; • 1 ♂, Shaanxi Province, Xianyang, Shimen Mt., National Forest Park, 2019. VII. 24, 35°04'27"N, 108°32'55"E, 1670 m, Qicheng Yang.

##### Diagnosis.

Eye bridge of three facet rows, contiguous. Dorsal and ventral surfaces of the wings are densely covered with dark brown microtrichia, only at apex of the veins on the dorsal surface covered with white microtrichia; vein R_5_ ending at wing apex. Ejaculatory apodeme 2.2× length of aedeagus. Parameres long and slender, pointed at the distal, 1.57× length of aedeagus. Gonostyle slender, with 2 setae distally. Surstylus with 27 tenacula distally, tenacula have three forms: short and straight, long and straight, long and curved.

##### Description.

(*N* = 1) **Male.** Body length 2.3 mm. Wing length 2.3 mm, width 1.2 mm. Head width 0.44 mm, length 0.4 mm; vertex 0.12 mm. Antennal 15 segments, scape length 0.12 mm, pedicel length 0.058 mm, width 0.066 mm; 1–13 flagellomeres: 0.12: 0.058: 0.095: 0.086: 0.086. 0.088: 0.88: 0.087:0.87: 0.09: 0.09: 0.09: 0.08: 0.069: 0.057. Palpomeres 1: 0.032, 2: 0.18, 3: 0.2, 4: 0.22.

**Figure 1.**
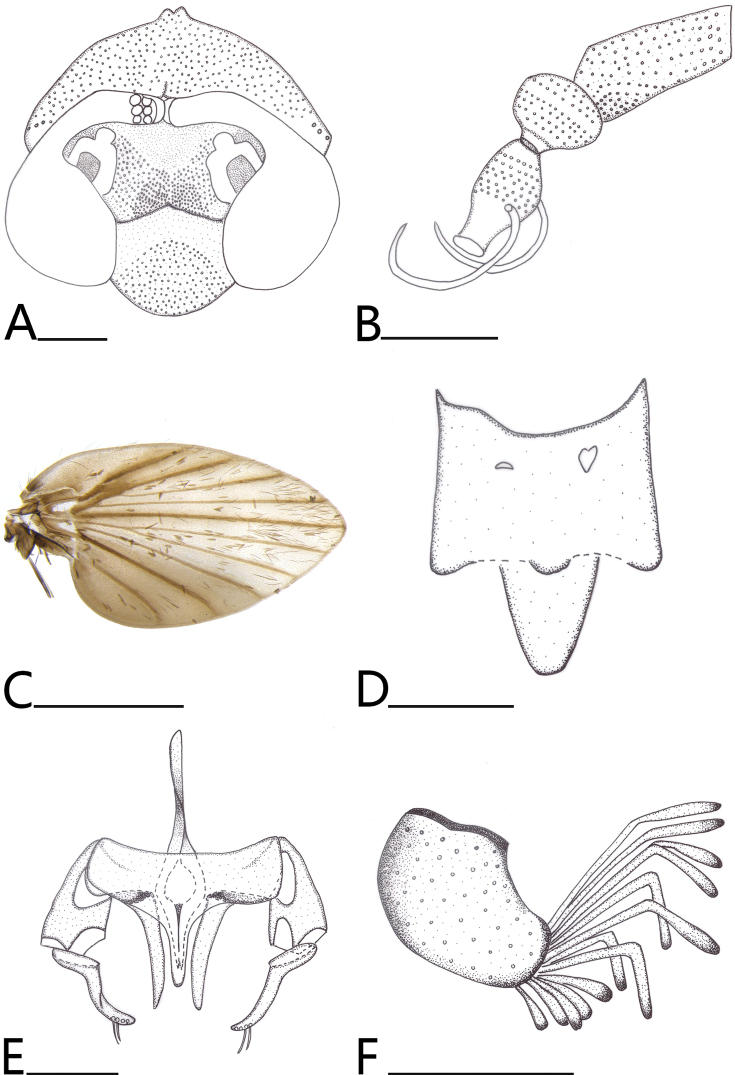
*Brunettia sinensis* sp. nov. A. Head; B. Antennae; C. Wing; D. Epandrium, dorsal view; E. Aedeagal complex; F. Surstylus, lateral view. Scale bars: 1 mm (C); 0.1 mm (A, B, D–F).

***Head*** (Fig. 1A) about the same length as width; vertex about 1/4 times length of head; with 4–6 ocular setae. Eye bridge of three facet rows, contiguous; interocular suture inverted Y-shaped; frontal scar patch concentrated at the middle half of the frontal and inner margin of the antennal socket, not extended to interocular suture. Antennae (Fig. 1B) 15 segments, scape cylindrical, distally widened; pedicel rounded, distally rather pointed; flagellomeres fusiform, swollen at middle and narrowed distally; two transparent ascoids, long, slightly curved, same width in all the length. Clypeus margin slightly U-shaped, clypeus scar patch more than half of clypeus, labellum bulbous. Palpal proportions 1:5.63: 6.25: 6.88

**Figure 2.**
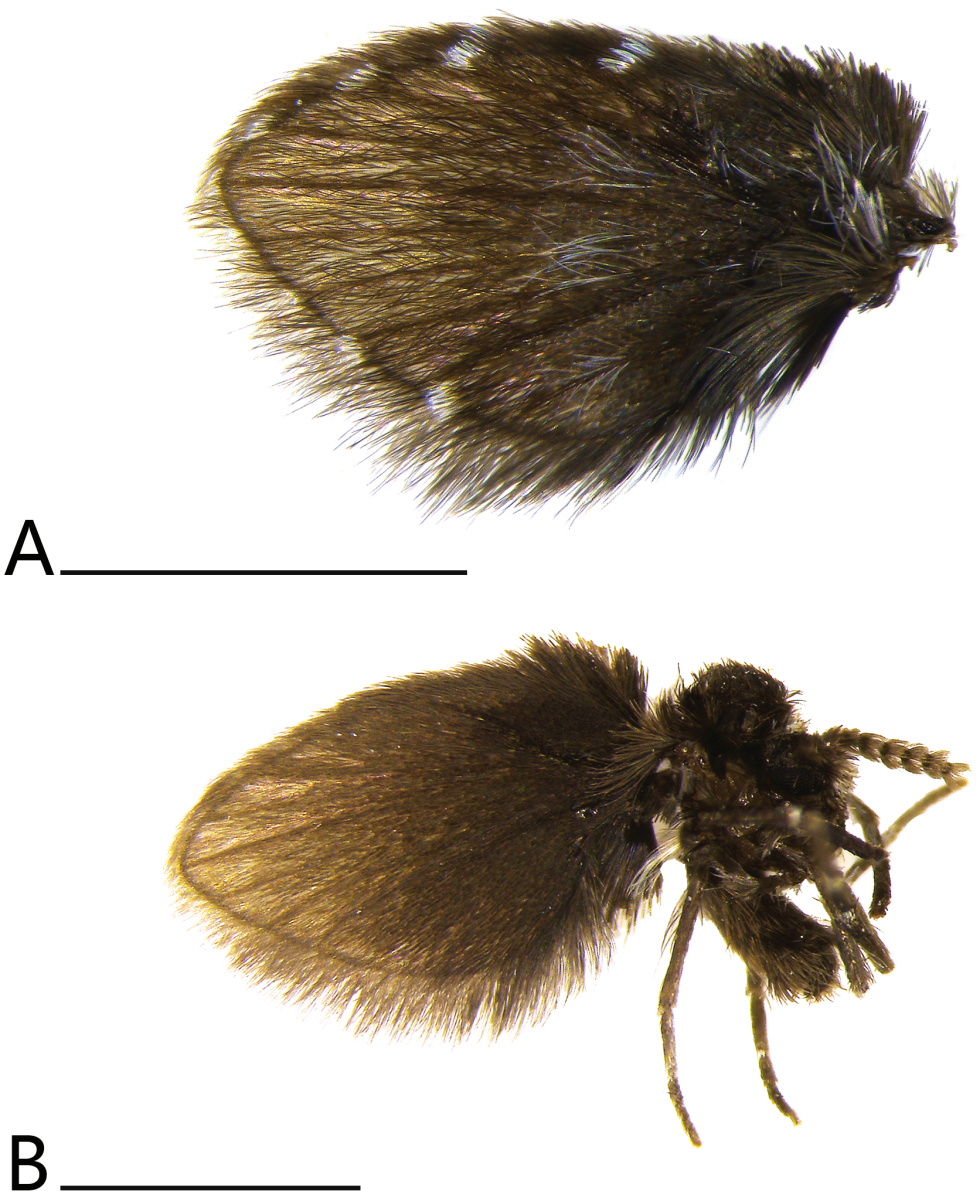
*Brunettia sinensis* sp. nov. A. Wing; B. Adult, lateral view. Scale bars: 1 mm.

***Thorax*.** Epimeron, anepisternum and katepisternum smooth, anepimeron half smooth and half setae. Wing (Fig. 1C) densely clothed with fine black setae, microtrichia forming white spots at the apex of the veins in dorsal view (Fig. 2A), but R_5_ at apex with white spot rather indistinct; at the middle with white, sparse microtrichia forming indistinct pale spots. Wing wide, veins brownish, covered with dark brown scales; humeral region expanded and dark brown at the margin; vein R_1_ rather wide, radial fork and medial forks complete, R_5_ ending at wing apex.

***Terminalia*** (Fig. 1D–F). Ejaculatory apodeme length 0.16 mm, rod-shaped, long and thin, obvious broad at apex in lateral view; ejaculatory apodeme 2.2× length of aedeagus. Aedeagus length 0.07 mm. Parameres long and slender, pointed distally, length 0.1 mm, about 1.57× length of aedeagus; basal of parameres length 0.079 mm; hypandrium wide, and the middle extension length exceeds the aedeagus. Gonocoxite rather wide and round, about 1.2× length of gonostyle; gonostyle slender, with 2 nearly transparent setae distally. Epandrium rectangular with two irregular pseudospiracular openings; hypoproct small, extended at the middle, tongue-shaped in dorsal view. Surstylus slightly broad, length 0.087 mm, width 0.059 mm in lateral view; surstylus with 27 tenacula distally, tenacula have three forms: 7 short and straight, 15 short and curved, 5 long and curved, contains 7 straight and 20 bend, tenacula length 0.04–0.098 mm, slightly oblate and darker distally.

**Female.** Unknown.

##### Distribution.

**China** (Fujian, Jilin, Shaanxi).

##### Etymology.

The specific name refers to China, referring to the new species distributed in the south and north of China.

##### Biology.

**Figure 3.**
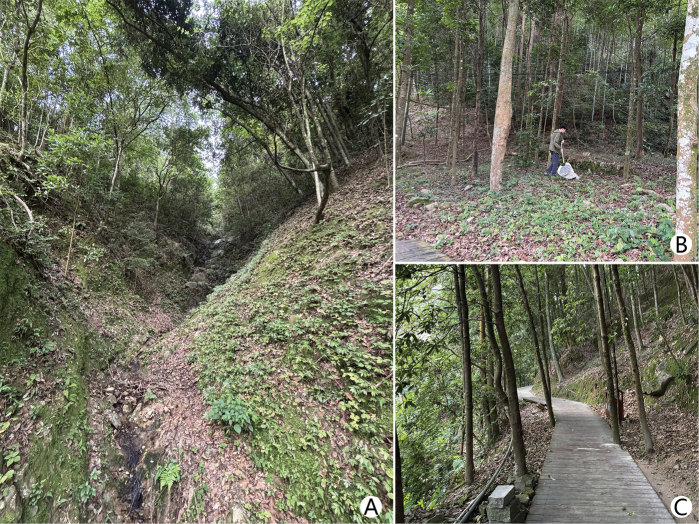
*Brunettia sinensis* sp. nov., specimen collection site in Fujian Province, Fuzhou, National Forest Park (photo by Miss Rong Huang). A. Habitat around the collection site; B. Shuailai Yang collecting specimens; C. Footpaths around the specimen collection site.

Specimens of this new species were collected through sweep netting in densely vegetated broad-leaved forests with abundant leaf litter and humus on the ground (Fig. 3A–C).

##### Remarks.

The new species resembles *B. spinistoma* Tokunaga & Komyo,1955 in the habitus, parameres long and slender, ejaculatory apodeme developed and long; but it can be separated from the latter by the following: eye bridge contiguous; ejaculatory apodeme and aedeagus 2.5× length of gonostyle; and the tenacula have three forms: short and straight, long and straight, long and curved. In *B. spinistoma* Tokunaga & Komyo, the eye bridge is minute; ejaculatory apodeme and aedeagus 2.0× length of gonostyle; and the tenacula have two forms, short and straight, long and curved (Tokunaga and Komyo 1955).

#### ﻿ Brunettia zunyiensis sp. nov.

Taxon classificationAnimaliaDipteraPsychodidae

E0BB6943-15EF-5FD6-A9E5-7445F5DCB6E0

https://zoobank.org/0DBFE56E-B17B-42E9-8AAA-36F53D52C68D

Figs 2A, B, 4A–F

##### Type material.

***Holotype*** • 1 ♂, **China**, Guizhou Province, Zunyi, Kuankuoshui National Nature Reserve, 28°14'28"N, 107°12'19"E, 2016.VII.22, 1147 m, leg. Malaise Trap. ***Paratypes***: • 1 ♂, same data as for holotype.

##### Diagnosis.

Eye bridge of three facet rows, separated by about 1 facet diameter. Wings humeral region obviously expanded and dark brown at the margin; R_1_ widened, R_5_ ending slightly below wing apex. Ejaculatory apodeme broad at the apex and rather curved, U-shaped. Parameres slender, pointed distally. Gonostyle slender, with 4 setae placed distally. Epandrium rectangular; pseudospiracular openings of epandrium oval-shaped; surstylus with 13–14 thick, short and straight tenacula distally, tenacula slightly oblate and darker distally.

##### Description.

(*N* = 1) **Male.** Body length 2.3 mm. Wing length 3.3 mm, width 1.8 mm. Head width 0.59 mm, length 0.5 mm, vertex 0.12 mm; antennal 15 segments, scape length 0.2 mm, pedicel: 0.083 mm, width 0.09 mm; 1–13 flagellomeres: 0.16, 0.13, 0.13, 0.14, 0.13, 0.12, 0.13, 0.14, 0.13, 0.12, 0.1, 0.085, 0.06. Palpomeres 1: 0.15, 2: 0.42, 3: 0.35, 4: 0.26.

**Figure 4.**
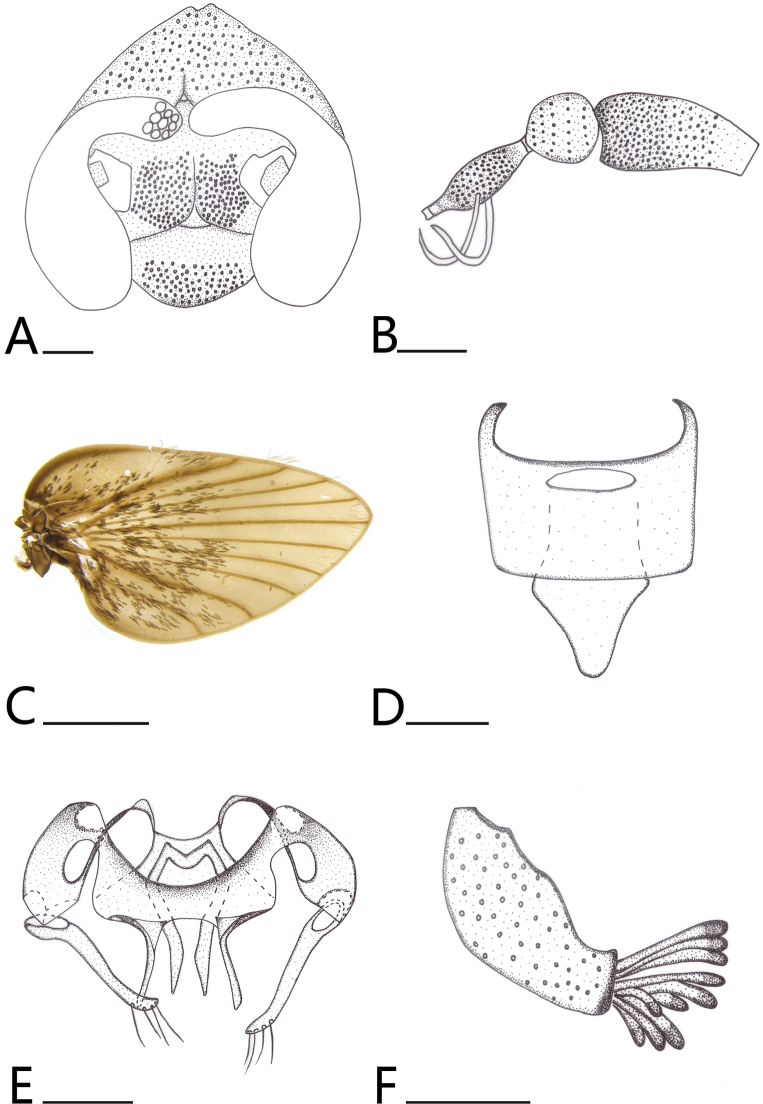
*Brunettia zunyiensis* sp. nov. A. Head; B. Antennae; C. Wing; D. Epandrium, posterior view; E. Aedeagal complex; F. Surstylus, lateral view. Scale bars: 1 mm (C); 0.1 mm (A, B, D–F).

***Head*** (Fig. 4A) head 1.2× wider than length; vertex about 1/5 times length of head; without obvious ocular setae. Eye bridge of three facet rows, separated about 1 facet diameters; interocular suture inverted Y-shaped; frontal scar patch concentrated at the middle, disappearing near the inner margin of the eye bridge and not extending to interocular suture. Antennae (Fig. 4B) 15 segments, scape cylindrical, 2× as long as the width, the distal slightly wider; pedicel about the same length as width, rounded, distal slightly pointed; flagellomeres fusiform, swollen at middle and narrowed distally; two ascoids transparent, long, slightly curved, same width in all the length. Clypeus margin slightly U-shaped, labellum bulbous. Palpal proportions 1: 2.52: 2.1: 1.56.

***Thorax*.** Epimeron with sparse setae, anepisternum and katepisternum smooth, anepimeron half smooth and half setae. Wing (Fig. 4C) brownish, broad with dark brown scales, humeral region expanded and vein C rather dark at the margin; vein R_1_ widened, radial fork and medial forks complete; vein R_5_ slightly below the top of the wing.

***Terminalia*** (Fig. 4D–F). Ejaculatory apodeme underdeveloped, very indistinct, broad at the apex and rather curved, U-shaped, and the middle is depressed. Aedeagus length 0.14 mm. Parameres length 0.094 mm, slender, slightly pointed distally. Gonocoxite wide and round, about 0.8× length of gonostyle; gonostyle rather long and slender, with 4 setae distally, setae near transparent. Epandrium rectangular; pseudospiracular openings of epandrium oval-shaped; hypoproct large, extended at the middle, slightly protruding near the epandrium, approximately tongue-shaped. Surstylus slightly slender, length 0.12 mm, width 0.083 mm from the lateral view. Surstylus with 13–14 tenacula distally, thick, short and straight, length about 0.042–0.067 mm, tenacula slightly oblate and darker distally.

**Female.** Unknown.

##### Distribution.

**China** (Guizhou).

##### Etymology.

The specific name“*zunyiensis*”refers to the type locality, Zunyi, in Guizhou Province.

##### Remarks.

The new species is similar to *B. napaea* Duckhouse, 1991 in the habitus, ejaculatory apodeme broad; epandrium rectangular; and the pseudospiracular openings of the epandrium oval-shaped, but it can be separated from the latter by the following: eye bridge of three facet rows; wing broad, 1.8× as long as wide; and the surstylus with 13 or 14 thick, short and straight tenacula. In *B. napaea* Duckhouse, the eye bridge has four facet rows; the wing is slender, 3.2× as long as broad; and the surstylus with 8 long tenacula (Duckhouse 1991).

## ﻿﻿Discussion

Thirty-six known species of *Brunettia* are distributed in the Oriental and Palaearctic regions. Nine species are known in India: *B. superestes* (Annandale, 1908), *B. albonotata* (Brunetti, 1908), *B. flavicollis* (Brunetti, 1911), *B. annandalei* (Brunetti, 1908), *B. argenteopunctata* (Brunetti, 1908), *B. atrisquamis* (Brunetti, 1908), *B. novemnotata* (Brunetti, 1911), *B. squamipennis* (Brunetti, 1908) and *B. panchpulaensis* Ipe, Ipe & Kishore, 1986; three species are known in Japan: *B. ishiharai* Tokunaga & Komyo, 1955, *B. lucidisquama* Tokunaga, 1959 and *B. spinistoma* Tokunaga, 1959; eleven species are known in the Philippines: *B. amoena* (Quate, 1965), *B. exulans* Quate, 1965, *B. hispida* Quate, 1965, *B. kibawa* Quate, 1965, *B. mateola* Quate, 1965, *B. mindanensis* Quate, 1965, *B. nubicola* Quate, 1965, *B. pallens* Quate, 1965, *B. parexulans* Quate, 1965, *B. recepta* Quate, 1965 and *B. yoshimotoi* Quate, 1965; six species are known in Malaysia: *B. albonotata* (Brunetti, 1908), *B. orchestris*, Quate, 1962, *B. brevifurca* Satchell, 1958, *B. pendleburyi* Satchell, 1958, *B. triangulata* Satchell, 1958 and *B. tormentosa* Satchell, 1958; and three species are known in Sri Lanka: *B. albohumeralis* (Brunetti, 1911), *B. albonotata* (Brunetti, 1908) and *B. uzeli* Ježek, 1996 (Annandale 1908; Brunetti 1908, 1911; Freeman 1951; Tokunaga and Komyo 1955; Satchell 1958; Tokunaga 1959; Quate 1962, 1965; Ipe et al. 1986; Huang and Chen 2001).

With the exception of three species distributed exclusively in Japan, *Brunettia sinensis* sp. nov. represents the sole species recorded within the Palearctic region. Occurring in both North and South China, this species is hypothesized to potentially have a widespread distribution across the country.

## Supplementary Material

XML Treatment for Brunettia sinensis

XML Treatment for Brunettia zunyiensis

## ﻿Additional information

### Use of AI

No use of AI was reported.

### Author ORCIDs

Shuai-Lai Yang https://orcid.org/0009-0002-6813-8949

Xin-Ming Yin https://orcid.org/0000-0003-3936-3557

Yu-Qiang Xi https://orcid.org/0000-0002-3285-8904

### Data availability

All of the data that support the findings of this study are available in the main text.
